# A double-edged sword?: Digitalization, health disparities, and the paradoxical case of e-pharmacy in Ghana

**DOI:** 10.1177/20552076251326224

**Published:** 2025-03-17

**Authors:** Shamshad Khan, Naessiamba Eab-Aggrey

**Affiliations:** 1 205267 Department of Communication, University of Texas at San Antonio, San Antonio, TX, USA; 2 Department of Communication, 3298 George Mason University, Fairfax, VA, USA

**Keywords:** E-pharmacy, online pharmacy‌, health disparities, Ghana, digital healthcare, qualitative research, ‌health communication

## Abstract

**Objective:**

With the ongoing push for greater digitalization of healthcare in low- and middle- income countries (LMICs), the larger questions around who will benefit most from such efforts and what elements of disparities and inequities may further be created or reinforced are often overlooked. This study was undertaken to assess a pioneering e-pharmacy initiative in Ghana that aimed to explore issues of access and disparities in relation to pharmaceutical services.

**Method:**

The study used a qualitative research design where semi-structured interviews were conducted virtually with 21 licensed community pharmacists recruited through purposive and snowball sampling techniques. The data were analyzed using inductive thematic analysis approach.

**Results:**

Pharmacists recognized the transformative potential of e-pharmacies, particularly in resource constrained regions that face issues of pharmacy and healthcare deserts. However, drawing on their experiential knowledge, they highlighted the paradoxes and challenges of promoting digitalization of healthcare in a country characterized by poor infrastructure, poverty, and multiple intersecting layers of inequities, as well as digital divides and low digital/health literacy. In the absence of adequate infrastructure, funding support and regulation, the possibility of local pharmacies, often the first point of care, being replaced by big corporations was feared. Participants also cautioned to steer the discourse of e-pharmacy away from access, pricing, and convenience to safety and quality.

**Conclusion:**

Digitalization of healthcare and e-pharmacies holds tremendous potential in the LMICs. However, such technological initiatives, if implemented without proper groundwork and adequate support, would run the risk of creating and exacerbating health disparities, especially in sub-Saharan Africa. A bottom-up approach, through grassroot engagement and implementation science, tethered to building safe, affordable, and equitable infrastructure and access to care will be essential for the success of e-pharmacy and other digitalization initiatives in the region and beyond. This research has direct implications for public health, policy, and pharmaceutical care.

## Introduction

The global landscape continues to transition into the digital age, marked by rapid changes in commerce, communication, and healthcare, which are increasingly apparent even in the “underdeveloped” regions of the world.^1^ The ongoing shift is predictable, particularly in the wake of the COVID-19 pandemic and given the push for digitalization of health by the World Health Organization (WHO), calling its member states to target healthcare digitalization by 2025.^2^ This initiative of the WHO has spurred low- and middle-income countries, like Ghana, to launch several digitalization initiatives across different sectors, including the telecommunications, and healthcare.^3,4^ The rapid digitalization of healthcare in Ghana is particularly exemplified by the initiation and thrust for expansion of online pharmacies at a national scale. In late 2021, the government of Ghana, in collaboration with the Pharmaceutical Society of Ghana, embarked on a journey to pilot 45 online pharmacies across the country on one platform.^5,6^ With promising pilot results, in July 2022, this initiative was further launched nation-wide as the National Electronic Pharmacy Platform (NEPP), with the goal of making pharmaceutical care more accessible to every Ghanian.^7,8^ This pioneering initiative is unique in the African region and is indeed commendable. However, with the uneven spread of technological progress in the country, the expansion of online pharmacies in Ghana, similar to other developing countries, warrants a careful scrutiny as it otherwise may exacerbate existing health disparities, potentially jeopardizing one of the United Nations’ Sustainable Development Goals (SDG) of providing an accessible, affordable, and equitable healthcare to all by 2030.

Online or e-pharmacy is increasingly popular as it offers an alternative and easy way for people to access healthcare products and services. Eysenbach (2001) defines e-health as any internet and technologically enhanced business or medical informatics that renders health services and information delivery.^9^ These technologies enhance and support health systems to improve care delivery and offer benefits to customers and pharmacists alike.^10^ E-pharmacy, as used in this study, is defined as any pharmacy that transacts part or all of its transactions online. As noted in several studies, e-pharmacy has its fair share of benefits and challenges, particularly in countries that are still building and expanding their infrastructure and regulatory measures. For instance, e-pharmacies come with benefits such as convenience, privacy, and easy access to medication at often competitive prices, which make them more attractive than the traditional store pharmacy.^3,11,12^ In addition, e-health technology has the potential to tackle stigma linked with certain health issues, including sexuality, that benefit from remote, online interactions with pharmacists.^13^

However, in regions with poor internet connectivity, some of these potential benefits are lost to large segments of population. Limited and intermittent access to the internet and unreliable electric power supply are often cited as the major barriers, making it challenging for e-health technology to offer its benefits to those living in areas with poor infrastructure.^3,14,15^ Furthermore, an emerging challenge of e-pharmacy is the lack of effective laws and regulations governing this industry, particularly in developing countries, where it is still in an early stage.^16–18^

While e-pharmacy promises an alternative and easy way for people to access healthcare products, it comes with a risk of potential misuse of medication, cascading into anti-microbial resistance and addictions, especially when e-pharmacies are not appropriately regulated.^18–21^

Here, it is important to briefly describe the concept of health disparity. Health disparity is defined as preventable differences in health outcomes that result from conditions or contexts that disadvantage people or groups from achieving their full social and economic potential.^22–24^ Such disparities have been linked to different kinds of disadvantages including lack of access to services and information, poverty, mental stress, poor resourced neighborhoods or areas, lack of transportations, inadequate health and digital literacy, and a host of other related conditions.^25^ Within the pharmacy context, a small but growing body of literature has foregrounded health disparities in pharmaceutical care, including how perceptions and awareness of pharmacists about existing disparities could influence their clinical practice.^26,27^ In particular, topics and issues such as ‘pharmacy deserts’, costs and access to medications, and lack of trusting relationships with pharmacists were found to be key challenges in mitigating health care disparities in relation to pharmaceutical care.^24,26,28,29^

However, there still remains a considerable gap in literature concerning disparities resulting from or related to current and ongoing expansion of e-pharmacies. Given the newness of this initiative, especially in much of the Global South where countries are also working toward their goals of achieving equitable, affordable, and universal access to health care (as part of SDGs), exploring experiences related to online pharmacies is extremely important.^1,2,16^ Unfortunately, much of the literature on e-pharmacy remains focused on the technological progress and possibilities, as well as on business and entrepreneurial logics (of expanding new frontiers of digital health economy), that nevertheless ignores important critical questions as to whose values, needs and interests are represented by these interventions, who may or may not benefit from them, and what elements of health disparities and inequities may further be created or reinforced.

The present study was designed to broadly explore these questions. In the wake of the initiation and expansion of e-pharmacy project in Ghana, our goal was to understand issues of equity and access to pharmaceutical care from the perspectives of the licensed community pharmacists. We particularly wanted to understand how the implementation of e-pharmacies might impact the nature and quality of their practice, and how they perceived issues of access and disparities pertaining to pharmaceutical services in different regions of Ghana. More specifically, the following research questions were explored: (1) How do local pharmacists perceive and experience the newly introduced e-pharmacy in Ghana? (2) What promises and challenges related to e-pharmacy are identified by the local pharmacists? (3) What are their recommendations for a more efficient and equitable electronic pharmacy system in Ghana? We argue that community pharmacists often work on the frontlines of care provision across the globe and especially in the Global South and have a rich understanding of the trade and the community they serve and work with. Drawing on their experiences and perspectives, with the help of qualitative research—which is significantly lacking in the field of digital health and pharmacy—has direct implications for public health and policy and pharmaceutical care for local, regional and global health and wellness.

## Methods

Using a qualitative research design,^30^ semi-structured interviews were conducted with licensed community pharmacists in Ghana to explore their experiences and perceptions about the pilot phase of e-pharmacy launched by the Pharmaceutical Society of Ghana.^5,13^ A qualitative research approach was found to be particularly suitable for the study as it allowed a detailed exploration into the experiences of the pharmacists as well as of their perceptions and apprehensions about online pharmacy. This study secured an institutional review board (IRB) approval in 2022 from the University of Texas at San Antonio (UTSA) with an IRB number of FY 21-22-93. Both authors were located at UTSA at the time of the study.

The research site for this study was Ghana, being a pioneer among sub-Saharan countries to initiate an online pharmacy nationally.^8^ The recency of this endeavor prompted the need to explore pharmacists’ views on this topic. Prior to 2018, the country was divided into ten main geographical regions that included: Western, Eastern, Central, Northern, Brong-Ahafo, Volta, Ashanti, Upper West, Upper East, and the Greater Accra regions. This list was expanded to 16 administrative regions in 2018, however, the licensed community pharmacists in our study continued to align their practice locations with the old ten regions, as outlined above.

### Data collection and sampling strategy

The data collection took place over the months of February and March 2022. For recruitment purposes, a broad inclusion criterion was adopted wherein all licensed retail community pharmacists practicing in Ghana were eligible to participate in the study, thereby excluding pharmacists without a valid license or practice at the time of data collection. The second author, who is a pharmacist from Ghana and trained in qualitative research as part of her master's degree, organized and completed the data collection for the study. The first step involved seeking permission to join the WhatsApp platforms for pharmacists belonging to the different regions in Ghana. To facilitate this process, key members and managers of the community pharmacists’ WhatsApp groups were approached and detail information about the study and the research team was provided and insights were sought. Once the approval was granted, more information about the study and the interviewer was shared on the WhatsApp platforms, along with an open invitation to pharmacists to participate. Those who responded with an initial interest, a follow-up message was sent with details about consent, confidentiality, and the process of virtual interviews. Participants were recruited using a purposive sampling technique and, specifically, using a combination of the maximum variation and snowballing sample techniques.^31^ In all, 26 pharmacists showed an interest to participate in the study. However, only 21 pharmacists finally participated as five interviews could not be completed due to poor internet connectivity. Besides, since data saturation was reached, we decided not to recruit any more participants.

Consent forms were sent to the participants prior to the interviews and any pending questions were clarified. Verbal consents were sought and secured at the start of the interview with the reinforcement about the voluntary nature of these interviews and the confidentiality aspects. All interviews were conducted virtually using the Zoom platform and lasted between 25 to 70 minutes. An interview guide was prepared with questions asking participants to share their thoughts about the newly launched online pharmacy, their personal experiences (if any), benefits and challenges that they anticipated, and other issues that they considered important to share on the topic. Follow-up questions were asked as needed to get an in-depth understanding of each participant's experiences and perceptions.

### Data analysis and study rigor

An inductive thematic analysis approach was undertaken to analyze the data.^32^ The interviews were transcribed verbatim and were then read and re-read by the authors to understand the emerging themes in the data. Interview data was inductively coded in several rounds, with finetuning of themes and patterns in each round. To protect the identity of participants, pseudonyms were used. This study was meticulously conducted, ensuring comprehensive documentation of all study processes securely stored for reference. Furthermore, journals were diligently maintained throughout the survey, capturing reflections on the process and participants.^13^

## Results

Of the 21 pharmacists who participated, there were nine females and 12 males, with most (85%) being in the range of 25–40 years and 38% being from the Greater Accra region (see Table 1 for more participant details). These socio-demographic findings are consistent with other research that have found the average age of pharmacists in Ghana to be in the range of 32–37 years^33–35^ and with a concentration of pharmacists in the Greater Accra region, as highlighted in Pharmaceutical Society of Ghana report (2022/2023) and other studies.^36,37^

**Table 1.** Demographics of participants.

Demographics	Frequency	%
Gender		
Female	9	42.9
Male	12	57.1
Age		
25–30	8	38.0
31–35	5	23.8
36–40	5	23.8
41–45	1	4.8
46–50	1	4.8
51–55	1	4.8
Location		
Ashanti region	1	4.8
Brong Ahafo	1	4.8
Central	3	14.3
Greater Accra	8	38.0
Northern	3	14.3
Volta	4	19.0
Western	1	4.8
Years of practice		
1–5 years	10	47.6
5–10 years	6	28.5
11–15 years	3	14.3
16–20 years	1	4.8
21–25 years	1	4.8
Experience with online pharmacy		
No	6	28.5
Yes	14	66.7
Not stated	1	4.8
Education		
Bachelor's degree	14	66.7
Master's degree	5	23.8
Other	2	9.5
Areas of practice		
Hospital pharmacy	5	23.8
Community pharmacy	8	38.0
Hospital and community	4	19.0
Community and academia	2	9.5
Regulatory pharmacist	1	4.8
Community and pharmaceutical representative	1	4.8
Positions		
Pharmacist	12	57.1
Pharmacy owner	4	19.0
Specialist pharmacist	2	9.5
Sales manager	1	4.8
Lecturer	1	4.8
Regulatory lead	1	4.8

Interviews with the participants revealed three overarching themes related to health disparities and differential access to e-pharmacy in different regions of Ghana. These were: (a) Digital divide and infrastructural inequities; (b) a trend toward a business model of pharmacy; and (c) the fall of community-based pharmacies. These themes unravel what may be described as the paradoxes of digitalization of pharmaceutical care—a double-edged sword—in a country like Ghana where large segments of areas and population are still steeped in social and structural inequities, despite considerable recent technological progress.

### Digital divide and infrastructural inequities

The digital divide in Ghana acts as a gatekeeper in that it grants access to resources to some while denying it to others. E-pharmacy provides clients with easy and private access to medications for their healthcare needs. However, this benefit remains unavailable to those with limited digital access or low digital literacy. For instance, while Ghana is experiencing an exponential growth in mobile phone technology, with increasing numbers having access to a mobile phone, this does not necessarily translate to high digital literacy. Pharm Jesse recounted an experience she had: “one person walked into my pharmacy, an elderly woman [with a phone], and she didn’t know what an App was…They are struggling with using WhatsApp. How will they use an app to order medicine.?”

In addition to low digital literacy, there is also the rampant issue of poor and intermittent internet connection in large parts of Ghana, which means that people living in those areas are unlikely to have consistent access to e-pharmacies. As was pointed out by Pharm Fiifi:

Areas where you don't have good internet connection, we have a difficulty accessing the service. And internet service in Ghana is just rated about 30% in terms of efficiency. So, if you live in an urban area, you have to spend well on your internet before you can get a high speed and then access, to be able to do this kind of thing, or else you spend more time waiting for some page to load and that will not be productive*.*

While the issue with internet connectivity largely affects people all over the country, expectedly, it is much worse in rural Ghana.

It is people who live around cities that are likely to get these[e-pharmacy] kinds of services, those who live in rural areas may not benefit because access to those areas are limited, road networks are bad. And the kind of volume of work that will flow into those rural areas tend to be so low that people may not be willing to invest so much money in going to those areas…. So, I believe that it will only serve people who are easy to reach, and not hard to reach. [Pharm Fifi]

The limited internet situation coupled with road infrastructural issues tend to make most e-pharmacies sideline these rural or hard-to-reach areas since they may not be as profitable as the urban center online orders. The paradox of access and infrastructure lies at the heart of the e-pharmacy dilemma in developing countries. While digital platforms promise greater accessibility to medications, the inadequacy of infrastructure in certain regions poses a significant barrier to realizing this potential.

Participants, who were pharmacy owners, and e-pharmacy pioneers, expressed their concern about infrastructural issues related to the delivery of medications. They noted that the postal service in Ghana, which could have been useful to promote e-pharmacy, has experienced a serious decline over the years and is now mainly patronized by a few corporate organizations.

Accordingly, Pharm Matt, a 51-year-old hospital pharmacist, argued for support from the government to make systems work and felt that inadequate infrastructure could seriously impede the expansion of the e-pharmacy pilot. He stated:

In terms of how systems are supposed to operate, the government infrastructure… the government is supposed to enable the space to be functional. They're supposed to enable electricity power to be stable. You know, during our conversation, power has gone off twice already. So, they are supposed to enable the power to be available. They are supposed to enable the communication, the online internet service provision is supposed to create an enabling environment.

Electrical power and internet were deemed essential for the smooth implementation of e-pharmacy in Ghana and many participants talked at length about the need to improve these amenities. Unfortunately, the state of electrical power supply and internet access is immensely poor in Ghana where only populations in major urban locations can be confident of the stable supply and that too only if they could afford it.

Road infrastructural inadequacies were also noted to be a big challenge in the operation of online pharmacies.

I think the number one challenge will be identifying locations… because most of those places [homes of clients], are not on the map. So, since we do [delivery] according to location, we ask that the patients send their location, so we can find the place, it may be a bit difficult to get to them…we know how our rural areas are…the routes to these places sometimes can be very uncomfortable… that ‘ll be one of the big challenges. [Pharm Jesse]

The [home] address system out here is also bad. So, trying to get someone's address would also be a problem. But I think they[govt.] are trying to solve that with the housing system. They're trying to number houses… so, I am thinking that they are one way or the other trying to solve this issue. [Pharm Sally]

One of the barriers to the smooth implementation of the e-pharmacy as stated above is the inadequate addressing system in the country and its implication for courier or postal services which may eventually affect the delivery of medications especially to rural areas or urban slums. It is important to note that about 45% of the urban population in Ghana lives in slums and more than 40% of Ghana is still rural.^38^ Thus, while e-pharmacy promises access to medication for all, some of these infrastructural issues and limitations, among others, will prevent large segments of population (almost half of the country) from having such access.

#### Trend toward a business model: issues of costs and pricing over safety

Another significant issue raised by most participants were around the costs associated with the operation of the e-pharmacy system. As Ghana's e-pharmacy sector is still in its early stages, logistical challenges of medication delivery could potentially inflate costs for pharmacists. This raises the dilemma of how pharmacists should address these expenses, whether by transferring them to clients or by absorbing them. Regardless of the chosen approach, participants unanimously agreed that the outcome would be unfavorable. Pharmacist Fiifi aptly encapsulated this sentiment when he remarked:

If you look at the process that your prescription has to go through before it gets to you, there are a lot of costs involved. Because anything at all you have to do packaging…. And you have to do delivery. So, you have to pass on all these costs to the patient. If you don't pass it on to the patient, it means that you have to bear the cost. And that doesn't make the process affordable. So, it would be more expensive getting the medication to the patient in his home than the patient walking to the pharmacy to pick up the medication on his own.

With the added costs of delivery, the pharmacists feared that the customers, unless they can afford, may not be as interested in using the e-pharmacy and may likely prefer to visit the pharmacy store instead. Pharm Yaa contextualized this issue within the socio-cultural milieu of Ghana:

Looking at Ghana that I am living in and the experience I've had, once you start asking people to pay for a delivery, and it sounds a bit on the high side, they would rather want to go through the traffic and the stress and come to the physical pharmacy, and buy the drug [laughs] so it defeats the whole purpose of even having the online shop…. [But]…For those who are in the high-end clientele? Yes. A lot of them. If they had an online option, they would just go for it. They don't care how much they are going to spend on delivery or whatnot.

Pharm Yaa's observations brings forth critical concerns for equity. If individuals from higher socio-economic backgrounds gravitate toward e-pharmacies, it could exacerbate disparities for those unable to afford medicine delivery costs. This situation disproportionately affects lower-income groups, potentially limiting their access to essential medications of good quality. With about 24.6% of the population in Ghana currently living below the poverty line, expansion of e-pharmacies without adequate infrastructural support could lead to serious issues of health disparities, especially if this also meant a concomitant loss of community-based pharmacies.^39^

On the other hand, as e-pharmacies offer easy access to medications, there is a potential for their misuse and overuse by people who can afford the costs of delivery. A few pharmacists noted that with medications being easily available online, and with pharmacy owners actively promoting sales of over-the-counter medications, it could result in overuse of cheap medicines potentially causing long-term harm to the customers.

Everybody seems to be talking about price…Pricing is the least important parameter…. You're talking about toxicity, you're talking about a product that [needs to be] well made to meet the standard, a product that has the right amount of the active ingredient, the product that can release the active ingredient… So pricing is not a good criterion for a customer to be selecting medicine online, otherwise, it will force the pharmacist as a businessperson to look for the cheapest source and not look at quality.[Pharm Edem]

These participants pointed to the risks of possible adverse effects that could result if patients without adequate information ended up consuming too much or wrong medications or when, in pursuit of profit, some pharmacies may sell medication online with substandard quality thereby compromising patient safety.

With the growing challenge of antibiotic misuse in Ghana,^20^ these pharmacists worried that as e-pharmacies become more common, there may be an even a bigger trend toward increased sale of antibiotics, in disregard to existing regulations, thereby widening the antimicrobial resistance spectrum in the country. They wanted to steer the discourse of e-pharmacy away from access, pricing, and convenience to safety and proper enforcement of regulations as otherwise, they feared, it would make e-pharmacy managers stock poor-quality products just to cut down on costs. This concern was summed up aptly by Pharm Laura:

Access to medication is important. But sometimes you're also thinking about the source. it depends on the legislation that they would want to put in place to ensure that we are getting credible medication from these online sources…safety should not be overlooked.

#### The fall of local community pharmacies: the first point of care

In Ghana, community pharmacies have been thriving for a long time as the first point of care for the local population. It is common for people to troop into the neighborhood pharmacy for various reasons, including seeking treatment for malaria, monitoring their blood pressure or blood sugar levels, seeking advice for their chronic illness, or getting treatment for STI's—even before they attempt to go to a hospital or a clinic—thus making the pharmacy one of the most sought out healthcare places in the country. Community pharmacists in Ghana are permitted by law to dispense, formulate, educate, and counsel their clients on their medication needs and to conduct “first aid treatment for simple ailments of common occurrence where it is not reasonably practicable for the patient to consult a medical practitioner or dentist.”^40^ Due to long commutes and waiting times at hospitals and clinics, many Ghanaians prefer to visit their community pharmacy for the initial consult, making it the first port of call for non-urgent medical conditions.^41–43^

With the introduction and promotion of e-pharmacy, however, local community pharmacies have come under pressure to either adopt the new system by investing more in their businesses or to stand to lose their customers in the long run. Several participants noted that small community pharmacy owners might not be able to sustain in the long run.

As a professional, I know that it will impact negatively my profession and the way I'm practicing, it will, because you are passing on cost to me that I don't have the backbone or the sustainable means to sustain that cost, I can't do it…the companies that will sign up [for online pharmacy], I tell you in future will be companies that have financial muscle, and you will now come and monopolize, they will import cheap medication and use that to serve as a medium to supply and that eventually may kill other community pharmacies… So, we also have to protect the community pharmacies that exists in the communities. And we can't do it if we don't get the framework right, to ensure that everybody can be on that [electronic] platform in a sustainable way. [Pharm Fifi]

So, we basically do the WhatsApp messages and calls [for online medication], which for my end is not so regular, like…we are the same people dispensing, so depending on the time I pick up the phone. That's when I will see the messages [for delivery] …. So, it is not something that is like our major practice… I think we will need to have someone [staff] dedicated for that. [Pharm Jesse]

What clearly comes out from the above quotes is the fear of big players coming in and wiping out small community-based pharmacies. Both Pharm Fiifi and Jesse articulated major concerns about the costs involved in operationalizing the e-pharmacy initiative including the cost of delivering medications, hiring more staff as well as of building of websites, and development of promotional materials. These pharmacists rightly anticipated the power play that may take place between the big pharmacies and the small pharmacies, creating a polarized front between these two groups. In other words, e-pharmacy was viewed as an initiative that would support the big players at the expense of the smaller ones. These pharmacy owners realized that the new initiative would require a change in the mode of operation from being the local neighborhood pharmacy to one catering to a larger clientele that would require adoption of a business model with substantial investment, which would not be easy for small pharmacy owners without necessary supports in place.

Some of the concerns around the possibility of small community pharmacies losing business leading to the monopolization of pharmacies in the hands of powerful players with financial capability, also stemmed from the lack of wider consultation before the introduction of the e-pharmacy initiative. For instance, Pharm Sally felt that if the success of the e-pharmacy pilot was important to the Pharmacy Council, it should have engaged the pharmacists on the ground:

I was like aah…they are trying to introduce e-pharmacy without engaging pharmacists! I don't know why they went about something like this…I don't know why they would do that. So, I have a problem…If you are trying to do something, if you are really serious about it, you engage the people who are already into that area…yes, maybe they[Council] engaged the (other) stakeholders, but these people are not the ones on the ground. So, you should have engaged those on the ground, most of us, let's say at the AGM, where most pharmacists in the country gather to do this.

Thus, the concerns about e-pharmacy initiative ranged from its economic implications and perceived partisanship toward big pharma leading to the consequent loss or disappearance of community pharmacies to the lack of consultations or inadequate engagement with pharmacists prior to its launch. The sentiment shared by several of these participants, although seemingly negative, primarily stemmed from the way the e-pharmacy policy was drafted that reflected a top-down approach. Without a meaningful and adequate consultation process, they felt alienated and believed the program would benefit the big players at the cost of community pharmacists who worked within the communities.

## Discussion

Online pharmacy is no new concept to the world. Over the years, numerous countries (especially in the West) have adopted and operated this system; however, for Ghana and most other developing countries, it is still in an infancy stage and yet to be widely accepted. The year 2021 particularly ushered Ghana into a new trajectory when the government encouraged pharmacists (through an e-pharmacy pilot project) to consider adopting e-pharmacies to reach out to clients, particularly those who did not have access to the conventional drug dispensing services in the country.^5^ In response, while some pharmacists were more in favor, others were in a dilemma, trying to weigh the pros and cons of this innovation. The current study was designed specifically to explore the perceptions of pharmacists about the promises and opportunities as well as challenges of the newly introduced e-pharmacy system against the backdrop of the larger social and structural contexts of Ghana.^13^

The NEPP in Ghana is a pioneering and one of its kind initiatives in the sub-Saharan region.^8^ Ghana, like other African countries, has struggled for long to ensure access to affordable pharmaceutical care for its citizens. The NEPP was designed as an innovative and centralized approach to digitizing the process of prescribing and dispensing of medicines to people in the country. The aim of NEPP is to “to serve as the nodal platform for originating, delivering, and regulating online pharmacy services” in Ghana.^44^ As an ambitious government-led innovative program, NEPP is closely observed by regulatory bodies in other African countries, with similar goals of digitization of healthcare, who aim to adapt this initiative to their local contexts. Thus, the experiment in Ghana has a much wider regional implication.

Studies suggest that e-pharmacy offers numerous advantages including easy access to medications, privacy in accessing pharmaceutical care as well as buying medications at a competitive pricing, among other conveniences.^1,3,11,13,26^ Access to medications and healthcare services is particularly a big challenge for people living in rural and remote areas in Ghana as the country faces issues of pharmacy and healthcare deserts.^28^ Pharmacists in our study noted the low pharmacy-to-patient ratio in rural communities that often creates conditions for self-acclaimed pharmacists, with no formal knowledge or degree, to practice dispensing of medications.

However, herein lies the paradox. Our findings revealed that while e-pharmacy initiative in a country like Ghana has the potential of greatly benefitting large masses of rural and other populations living away from cities and well-resourced areas, the real benefits can only be reaped when structural inequities in the country have been addressed.^4^ Our study findings indicated several infrastructural challenges as well as issues of digital and health literacy that would impede a wide-scale adoption of this electronic platform. Pharmacists shared how unreliable electric power, poor internet connectivity, lack of adequate road infrastructure and reliable courier services, would create hurdles in the adoption and expansion of the electronic pharmacy system in the country. Similar findings have been noted in other research studies in the African continent where implementation of any electronic health-related system faced significant challenges due to poor electricity supply, lack of constant internet connectivity as well as high costs of internet services, and inadequate funding.^14,15,28^ Furthermore, limited resources available to most local pharmacies, including lack of dedicated pharmacy staff to handle online orders, was found to be an important practical constraint in the wide-scale implementation of electronic pharmacies, both in our study as well as in others.^45^ Relying on existing staff to cater to both store and online customers is often a reality for small pharmacy owners, but it could be a major hindrance for efficient service delivery as well as for the sustainability of e-pharmacy in the long run.

Digital technology is often closely associated with existing inequities that get reflected in digital divides at multiple levels, from physical access to hardware (computers, phones, etc.) to internet connectivity to digital literacy and empowerment, making it a multidimensional phenomenon that further intersects with people's race, age, gender, socio-economic status, geographical location and other structural realities. This has made digital inclusion one of the most sought-out goals for policymakers, particularly in the developing world, linking it with social inclusion and equity. A study that reviewed Ghana's goals and strategies for digitization and digital inclusion argued the need for indigenizing the approach to ensure better reception and commitment at the grassroots level that would ultimately result in intended outcomes.^46^ E-pharmacy in Ghana is currently at the awareness and persuasion stages where pharmacists have been introduced to the concept and are being persuaded by the pharmaceutical regulatory board, the pharmacy council, to join this initiative. Launched in 2022, to date, NEPP has over 200 online pharmacies onboard.^7,44^ While the progress is impressive, in terms of numbers, it only accounts for 5% of the existing pharmacies in Ghana and is responsible for only 1% of pharmaceutical sales in the country.^44^ Beyond numbers, the adoption pattern has been uneven with heavy concentration in the Greater Accra region, while northern regions, with poor infrastructure and digital literacy, are inadequately represented.^7^ Besides the regional infrastructural disparities, it was observed that even some well- resourced regions (like the eastern and central regions) had low levels of participation in e-pharmacy that could be due to poor awareness about this initiative.

Studies have noted that successful creation and implementation of e-pharmacy system requires an in-depth understanding of the socio-economic environment of the region to be able to anticipate and address potential barriers, be it related to technology or infrastructure or economic factors.^12^ Our study findings highlight the need for real engagement with community pharmacists, those working with the communities and well acquainted with ground realities, for successful adoption and expansion of NEPP.

Community pharmacists, as frontline healthcare providers and often as the first point of care, face the incredible challenge as well as the opportunity to develop complex clinical relationship with a diverse group of patients.^13^ Being often the only option for timely healthcare in rural or remote areas of the country, pharmacists are uniquely positioned not only to provide quality and equitable care but also to offer ways to address broader health and social disparities. It is through tapping this group, for their knowledge, experience and recommendations, that a country like Ghana could use innovative e-pharmacy initiative as a tool to achieve goals of universal access to health and pharmaceutical care. However, this cannot be achieved by technological advancements alone. It is attainable only through political will and government support, from building better infrastructure, house addressing system, reliable courier services to funding and training of small pharmacy owners that would close the health equity gap.

Digitalization of healthcare and electronic pharmacies are the future of countries working toward better health for all. However, such initiatives, if implemented without proper groundwork and adequate supports in place, would run the risk of worsening the health disparities in the region, thereby making their goals futile right from the start. As others have also noted, “Ghana's case demonstrates that grassroots engagement, as well as leadership from government…. will be key to the successful delivery of digital inclusion at all levels.”^46^ E-pharmacy could be a game-changer with tremendous transformative potential particularly for resource constrained regions but requires careful consideration and strategies for its success.

This study had a few limitations that need to be considered. First, being a qualitative study, it would have been ideal if the interviews were conducted in-person, allowing for face-to-face interaction between the interviewer and the interviewee, which would have helped with better rapport building as well as adding to the richness of data through an observation of social cues during the conduct of the interviews. However, given the focus of the study—that is, digital health and pharmacy—virtual interviews did add to the understanding of the experience of digitalization and the use of internet technology in the daily lives of the Ghanaian. Moreover, virtual data collection allowed for a more diverse sample that was not restricted to a specific geographical locality. Another limitation is the broad exploratory nature of this study that aimed to understand the range of perspectives held by community pharmacists about the expansion of online pharmacy in Ghana. As an early study in this area, while the exploratory nature was important and needed, as time has progressed since the introduction of NEPP, specific angles may be further investigated in a more detailed manner. Future research, by adopting a mixed-method approach, can target a bigger and diverse sample that goes beyond the perspectives of only community pharmacists to include patients as well as policymakers and other stakeholders. This would allow an in-depth understanding of how digitalization and e-pharmacy can be realistically implemented as well as how it can be integrated with existing traditional pharmacy care to maximize equitable distribution of the benefits to large sections of populations in Ghana and in other African countries.

## Conclusion

The exploration of online pharmacy reveals a nuanced landscape marked by both promise and peril. While it offers undeniable conveniences, it also introduces a corresponding set of challenges and disruptions, particularly in nascent markets like Ghana. The paradox of progress is evident: as e-pharmacy strives to revolutionize healthcare accessibility and delivery, it simultaneously threatens to worsen health disparities and result in poor quality and access to healthcare. As the pharmaceutical industry continues to evolve in the digital age, stakeholders must grapple with this paradox, striving to harness the benefits of innovation while safeguarding against its unintended consequences. Only through a steadfast commitment, political will, and community engagement can we fully realize the transformative potential of e-pharmacy while mitigating its inherent risks.

## Supplemental Material

sj-pdf-1-dhj-10.1177_20552076251326224 - Supplemental material for A double-edged sword?: Digitalization, health disparities, and the paradoxical case of e-pharmacy in Ghana Supplemental material, sj-pdf-1-dhj-10.1177_20552076251326224 for A double-edged sword?: Digitalization, health disparities, and the paradoxical case of e-pharmacy in Ghana by Shamshad Khan and Naessiamba Eab-Aggrey in DIGITAL HEALTH
